# A comparative study of efficacy of esmolol and fentanyl for pressure attenuation during laryngoscopy and endotracheal intubation

**DOI:** 10.4103/1658-354X.76473

**Published:** 2011

**Authors:** Shobhana Gupta, Purvi Tank

**Affiliations:** *Department of Anaesthesiology, Medical College, Jamnagar, Gujarat, India*

**Keywords:** *Esmolol*, *fentanyl*, *laryngoscopy endotracheal intubation*, *pressure response*

## Abstract

**Objective:**

To compare the effectiveness of single bolus dose of esmolol or fentanyl in attenuating the hemodynamic responses during laryngoscopy and endotracheal intubation.

**Methods:**

Ninety adult ASA I and ASA II patients were included in the study who underwent elective surgical procedures. Patients were divided into three groups. Group C (control) receiving 10 ml normal saline, group E (esmolol) receiving bolus dose of esmolol 2 mg/kg and group F (fentanyl) receiving bolus dose of fentanyl 2 *µ*g/kg intravenously slowly. Study drug was injected 3 min before induction of anesthesia. Heart rate, systemic arterial pressure and ECG were recorded as baseline and after administration of study drug at intubation and 15 min thereafter.

**Results:**

Reading of heart rate, blood pressure and rate pressure product were compared with baseline and among each group. The rise in heart rate was minimal in esmolol group and was highly significant. Also the rate pressure product at the time of intubation was minimal and was statistically significant rate 15 min thereafter in group E.

**Conclusion:**

Esmolol 2 mg/kg as a bolus done proved to be effective in attenuating rises in heart rate following laryngoscopy and intubation while the rise in blood pressure was suppressed but not abolished by bolus dose of esmolol.

## INTRODUCTION

Stress response under anesthesia has been universally recognized phenomenon which may be in the form of endocrine or autonomic disturbance. The pressure response to laryngoscopy and endotracheal intubation in form of tachycardia, hypertension and arrhythmias may be potentially dangerous. These changes are the maximum at 1 minute after intubation and last for 5-10 min.[1]

There is substantial evidence that, laryngoscopy and intubation is accompanied by a considerable increase in heart rate and arterial blood pressure. These changes are usually of short duration and well-tolerated by patients in the absence of cardiovascular disease or disturbed intracranial pressure homeostasis. In these conditions, an increase in blood pressure may lead to complications, including arrhythmias, myocardial ischemia, increase in intracranial pressure and rupture of cerebral aneurysms.[2,3]

Various methods of attenuation of response to laryngoscopy and intubation are still in search from the date of its recognition. Several studies have been made in order to attenuate these haemodynamic response to laryngoscopy and intubation. Many drugs also have been used for the same purpose.[4–10]

Esmolol is an ultra-short acting β-1 adrenergic blocker. It has predominant effect on β-receptors and possesses no significant membrane stabilizing activity. It has rapid onset and a short duration of action.[11–12]

Fentanyl is a phenylpiperidine of the 4-amino piper dine series, structurally related to, but not derived from pethidine.[13]

The aim of this study is to do a comparative study of esmolol and fentanyl in attenuating the pressure response during laryngoscopy and intubation.

## METHODS

After taking permission from hospital ethics committee and with the patients’ consent, we studied 90 patients of either sex weighing 35-60 kg, aged between 15-55 years were included in the study. All the patients were belonging to ASA grade I and II and were scheduled for elective surgical procedures.

Patients with predicted difficult intubation, hypertension, ischemic heart disease, compensatory tachycardia, baseline pulse <60 bpm, baseline systolic B.P. <100 mm Hg, chronic obstructive airway disease, on medicines with cardiovascular effects were and excluded from the study.

All patients were premedicated with 0.2 mg i.v. injection glycopyrrolate 10 min before surgery. Pulse, bloodpressure, SPO_2_, ECG were recorded before as well as after premedication. Pulse, systolic and diastolic blood pressure, oxygen saturation, were monitored continuously and recorded before premedication, after premedication and after intubation at 1, 3, 5, 15, 30, 45, 60, 75, 90 minutes and postoperatively. In group E and group F all these parameters were also recorded after drug i.e., esmolol and fentanyl, respectively.

Patients were divided into three groups:

**Table:** 

Group C:	Normal saline was given. (Control)
Group E:	Injection esmolol 2 mg/kg i.v. 3 min before laryngoscopy and intubation, over 30 seconds.
Group F:	Injection fentanyl 2 µg/kg i.v. 3 min before lar yngoscopy and intubation, over 30 seconds.

After preoxygenation and 3 min after the administration of the study drug, induction was done with injection thiopentone sodium 5 mg/kg and injection suxamethonium 1.5 mg/kg. Laryngoscopy and endotracheal intubation was performed 90 second after the administration of succinylcholine. In all the groups intubation was done with Macintosh curve blade with in a period of 15 seconds. Failure to intubate in this period and difficult intubation cases were excluded from this study. After confirming the position of the ET tube and fixing it anesthesia was maintained with 33% O_2_ and 66% N_2_O. Injection vecuronium was used as a muscle relaxant. Pulse, systolic and diastolic blood pressure, O_2_ saturation and ECG were monitored continuously and recorded at timely interval. At the end of the surgery all patients were reversed by using injection Neostigmine 0.05 mg/kg and injection glycopyrollate 8 µg/kg intravenously. Patients were then shifted to anesthesia recovery room and monitored for complications like pain, nausea, vomiting, respiratory depression, hypertension, hypotension, bradycardia, drowsiness and rigidity.

For statistical analysis of data within the groups, paired Students ‘*t*’ test was used while for comparison between groups unpaired ‘*t*’ test was used. Results were considered statistically significant for *P* values *P*< 0.05.

## RESULTS

Cases were selected from different specificity (general surgery, gynecology, orthopedics, ENT). The demographical data was compared among the three groups. No statistically significant difference between the groups was observed with respect to age, gender or weight.

Table 1 shows demographical data of all three groups. In group C mean age of patients was 31.3±2.38, group F was 32.83±10.5 and group E was 32.66±3.99. In group C there were 16 females and 14 males with a ratio of 1:1.14, group F there were 14 females and 16 males with a ratio of 1:0.87, group E there were 17 females and 13 males with a ratio of 1:1.30. Weight distribution (in kgs) in group E was 53.73±2.64, in group F was 54.70±4.35 and group C was 54.06±2.64.

**Table 1:** Demographical profile of the study group

Patient characteristics	Ratio of mean (SD)		
	Group E	Group F	Group C
Sex (M:F)	1:1.31	1:1.08	1:1.43
Age (years)	31.13	32.83	32.66
Bodyweight (kg)	54.06	54.70	53.73

**Figure 1:**
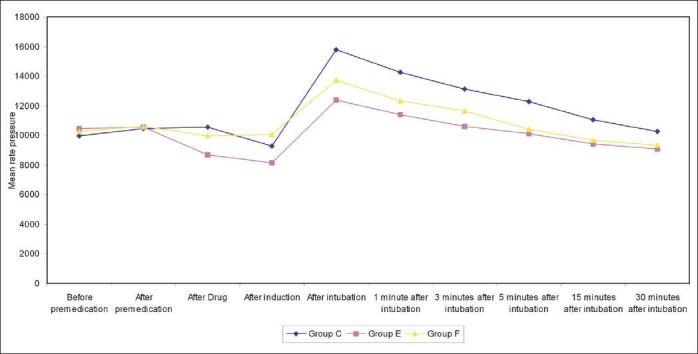
Graph showing comprehensive changes in rate pressure product

**Figure 2:**
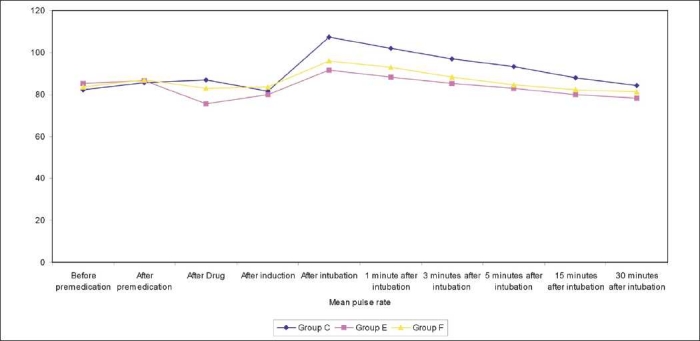
Graph showing changes in mean pulse rate

**Figure 3:**
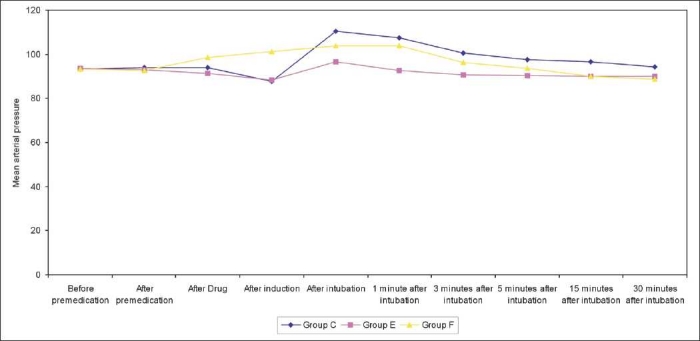
Graph showing comprehensive changes in mean arterial pressure

Table 2 shows change in mean pulse rate and mean arterial pressure and Table 3 shows in rate pressure product, in all three groups compared with their respective preinduction value at different stages [Figures 1–3].

**Table 2:** Changes in the mean pulse rate and mean arterial blood pressure in all three groups

Time		Mean pulse rate			Mean arterial blood pressure		
		Group C	Group E	Group F	Group C	Group E	Group F
Before premedication	Mean	82.2	85.2	83.6	93.36	93.73	93.29
	Range	72-92	68-109	64-110	83-100	85-102	83-105
	S.D.	±5.47	±7.60	±10.37	±5.41	±4.52	±5.66
After drug	Mean	86.8	75.53	82.93	94.10	91.45	98.69
	Range	74-96	60-84	64-98	84-104	82-97	83-97
	S.D.	±5.05	±6.76	±8.95	± 3.93	±3.74	±4.68
After induction	Mean	81.53	79.73	83.53	87.85	88.24	101.06
	Range	72-92	80-104	62-94	77-95	82-90	92-105
	S.D.	±5.01	±7.73	±4.32	±4.47	±3.92	±3.03
After intubation	Mean	107.33	91.66	95.86	110.56	96.70	103.88
	Range	98-116	86-104	88-104	100-112	92-103	93-110
	S.D.	±4.82	±5.01	±4.69	±3.24	±3.32	±3.60
1 minute after intubation	Mean	101.8	88.33	92.86	107.47	92.76	99.85
	Range	96-108	76-100	86-100	96-107	93-100	87-101
	S.D.	±4.34	±5.28	±3.95	±3.09	±3.11	±3.61
3 minutes after intubation	Mean	97	85.33	88.2	100.68	90.76	96.32
	Range	90-106	76-94	82-94	94-106	93-97	87-101
	S.D.	±4.44	±4.61	±3.45	±3.02	±1.83	±3.55
5 minutes after intubation	Mean	93.33	83.00	84.66	97.46	90.49	93.69
	Range	82-100	74-88	80-90	93-102	89-96	83-101
	S.D.	±4.82	±3.88	±3.25	±2.67	±1.97	±3.43
15 minutes after intubation	Mean	87.93	79.93	82.13	96.66	90.11	90.16
	Range	80-100	68-88	76-90	86-99	82-94	82-97
	S.D.	±4.59	±4.31	±3.10	±3.20	±3.65	±3.85

**Table 3:** Changes in the rate pressure product in all three groups

Time		Group C	Group E	Group F
Before premedication	Mean	9946.26	10431.4	10241.1
	Range	7920-1440	7920-11440	6784-14960
	S.D.	±897.60	±1286.2	±1703.33
After drug	Mean	10558.4	8672.4	9986.13
	Range	8288-11760	6572-10400	7040-12740
	S.D.	± 815.81	±1017.2	±1388.21
After induction	Mean	9269.2	8150.06	10039.67
	Range	8000-10836	6572-9826	10080-14144
	S.D.	±635.22	±863.95	±914.31
After intubation	Mean	15772.27	12396.7	13724
	Range	12740-19560	9980-14144	9382-14560
	S.D.	±1233.88	±941.03	±2223.34
1 minute after intubation	Mean	14269.93	11414.93	12348.8
	Range	12480-16200	9600-1300	10800-14016
	S.D.	±939.63	±861.43	±734.11
3 minutes after intubation	Mean	13134.8	10590.13	11651.6
	Range	11700-15180	9120-11960	9900-12521
	S.D.	±903.32	±746.75	±1680.80
5 minutes after intubation	Mean	12258	10129.93	10419.73
	Range	10080-13720	9120-11440	8692-11696
	S.D.	±910.28	±645.141	±663.83
15 minutes after intubation	Mean	11043.07	9403.06	9663.6
	Range	9600-12600	7208-10584	8360-11440
	S.D.	±4.59	±754.48	±668.52

The increase in heart rate at intubation was seen in all the three groups the baseline value. But the rise was minimal in group F and group E as compared to group C, which was statistically significant (*P*<0.05). Also, only in the group E, there was no significant rise at any time interval (*P*<0.001). These changes were significant up to 15 min postintubation [Table 4].

**Table 4:** Changes in heart rate at various time interval (mean ± SD)

Groups	After drug	After induction	After intubation	1 min after intubation	3 min after intubation	5 min after intubation	15 min after intubation
Group E	75.53±6.76	79.73±7.73	91.66±5.01	88.33±5.28	85.33±4.61	83.00±3.88	79.33±4.31
*P* value (ctrl/E)	0.042*	<0.05*	<0.001**	<0.001**	<0.001**	<0.001**	0.001**
Group F	82.93±8.95	83.53±4.32	95.86±4.69	92.86±3.95	88.2±3.45	84.66±3.25	82.13±3.10
*P* value (ctrl/F)	<0.041*	0.270	<0.001**	<0.001**	<0.001**	<0.01*	<0.01*

* = Significant,

** = Highly significant

Compared with baseline value, systolic blood pressure, diastolic blood pressure and mean blood pressure were increased in all the three groups after laryngoscopy and intubation and at different time intervals. The increase in mean arterial pressure was least in group E and maximal in group C. The rise was highly significant immediately after intubation in group C (*P*<0.001) and significant from (*P*<0.05) in group F while was not significant in group E (*P*>0.005) [Table 5].

**Table 5:** Changes in mean arterial pressure at various time interval (mean ± SD)

Groups	After drug	After induction	After intubation	1 min after intubation	3 min after intubation	5 min after intubation	15 min after intubation
Group E	93.73±4.52	88.24±3.92	96.70±3.32	92.76±3.11	90.76±1.83	90.49±3.65	90.11±3.64
*P* value (ctrl/E)		<0.001**	<0.0001**	<0.0001**	<0.0001**	<0.001**	0.001**
Group F	93.29±5.66	101.06±3.03	103.88±3.60	99.85±3.61	96.32±3.55	93.69±3.43	90.16±3.85
*P* value		0.0001**	<0.0001**	<0.0005**	<0.0001**	<0.001**	<0.05*

* = Significant,

** Highly significant

The changes in MAP were significant up to 15 min postintubation after which the systolic blood pressure, diastolic blood pressure and mean arterial pressure declined gradually and reached to baseline levels after 15 min of laryngoscopy and intubation in all the three groups. The RPP was calculated as the product of heart rate and systolic arterial pressure. In our study the RPP during intubation revealed a highly significant (*P*<0.001) increase in group C and group F, whereas the increase was insignificant in group E. These changes were highly significant up to 15 min postintubation (*P*<0.001).

While comparing group F to group E these was highly significant (*P*<0.001) increase in RPP in group F at the time of intubation and was statistically significant (*P*<0.01) at all the instances.

Table 3 is showing the comprehensive changes in the mean rate pressure product in all three groups. RPP is calculated as: RPP=SAP×SR. The mean RPP in group C, raise from 10136.67 ±1893.8to15772.27 ± 635.22 with a rise of 5636, which was highly significant than in group E, where the difference in the value of mean RPP after premedication and after intubation was 1508 while the same was 2103 in group F. These changes remain significant even after 15 min of intubation. The rise in mean RPP was least in group E and highest in group C. the changes were seen significant in group E compared to group C, even after 30 min of intubation.

## DISCUSSION

The pressure response to laryngoscopy and endotracheal intubation in form of tachycardia and hypertension occurs frequently; even α-adrenoreceptor blockade minimizes increases in heart rate and myocardial contractility (primary determinants of O_2_ consumption) by attenuation effects of increased adrenergic activity. This is particularly derivative in patients with IHD.[12–13]

More attention is given to the use of selective β-adrenergic antagonists to prevent the reflex sympathoadrenal discharge-mediated tachycardia and hypertension during procedures of laryngoscopy and endotracheal intubation and these include esmolol.[14]

Esmolol has been used in various bolus does or in an infusion form. Esmolol, 2 mg/kg, as a single bolus successfully attenuated the pressure response. There was minimal increase in heart rate than the other group but the blood pressure showed a rise although it was less than other groups after laryngoscopy and endotracheal intubation.[15]

Again our study correlates with the study of Liu Philip *et al*. who used esmolol infusion to control hemodynamic responses associated with intubation. They found significant decreases in RPP prior to induction and postintubation the increase was 50% less in the esmolol-treated patients compared to the placebo group.[16]

Christopher *et al*. used esmolol 1-2 mg/kg and concluded that the increase in heart rate and blood pressure associated with laryngoscopy and endotracheal intubation were significantly lower in comparison to the control group.[17]

Sabahat *et al*. used esmolol 1 mg/kg and concluded that esmolol partially attenuated the hemodynamic response but did not abolish it completely. Esmolol in bolus doses 100 mg and 200 mg attenuates tachycardia and hypertension after tracheal intubation.[18]

Esmolol group did not reveal any rhythm abnormality. No ST segment changes were seen in any patients.

Narcotics may block afferent nerve impulses resulting from stimulation of the pharynx and larynx during intubation

Fentanyl has also been used in different doses varying from 2 to 15 µg/kg to blunt haemodynamic responses to laryngoscopy and endotracheal intubation. Low doses of fentanyl, 2 µg / kg were used in our study and the efficacy was compared with esmolol group.

It was found that with fentanyl, 2 µg/kg elevation of heart rate and blood pressure after intubation was lower than control group, although not statistically significant.

Yushi *et al*. in his study concluded that 2 µg/kg fentanyl suppresses the hemodynamic response to endotracheal intubation more than the response to laryngoscopy.[19]

It was shown that supplementation of anesthetic induction with fentanyl 2 µg/kg significantly attenuated the increase in heart rate, arterial pressure and rate pressure product after laryngoscopy and intubation, and fentanyl 6 µg/kg completely abolished pressure responses.[20]

Doses of fentanyl that are low enough to same little postoperative respiratory depression significantly blunt postintubation hypertension when used as adjoins to thiopental. This was demonstrated in a study conducted by Donald E. Martin *et al*. who used fentanyl, 8 µg/kg in patients undergoing major vascular surgery.[20–21]

Low doses of fentanyl were employed because a large dose was lead to muscular rigidity, bradycardia, nausea and vomiting. Large doses may also cause postoperative respiratory depression; especially in surgery with short duration of less than 1 hour.[22] McClain *et al*. reported apnoeic episodes in four out of seven patients who received 3.2-6.5 µg/kg fentanyl.[23]

## CONCLUSION

From the present study it is evident that both esmolol in a bolus dose of 2mg/kg and fentanyl in bolus dose of 2 µg/kg before induction of anesthesia are effective in attenuating the hemodynamic responses to laryngoscopy and endotracheal intubation like heart rate and rate pressure product.

But only esmolol provided consistent and reliable protection against increases in both heart rate and systolic blood pressure accompanying laryngoscopy and endotracheal intubation.

No evidence of any myocardial insult was seen in any of the patients in any group in our study.

It is advisable and safe to use esmolol in patients who are prone to have exaggerated responses of cardiovascular system during laryngoscopy and intubation.
